# Genetics-Based Classification of Filoviruses Calls for Expanded Sampling of Genomic Sequences

**DOI:** 10.3390/v4091425

**Published:** 2012-08-31

**Authors:** Chris Lauber, Alexander E. Gorbalenya

**Affiliations:** 1 Molecular Virology Laboratory, Department of Medical Microbiology, Leiden University Medical Center, 2333 ZA Leiden, The Netherlands; Email: c.lauber@lumc.nl; 2 Faculty of Bioengineering and Bioinformatics, Lomonosov Moscow State University, 119899 Moscow, Russia

**Keywords:** genetics-based classification, taxonomy, DEmARC, filoviruses, ebolavirus, virus species, genetic diversity, pairwise evolutionary distances

## Abstract

We have recently developed a computational approach for hierarchical, genome-based classification of viruses of a family (DEmARC). In DEmARC, virus clusters are delimited objectively by devising a universal family-wide threshold on intra-cluster genetic divergence of viruses that is specific for each level of the classification. Here, we apply DEmARC to a set of 56 filoviruses with complete genome sequences and compare the resulting classification to the ICTV taxonomy of the family *Filoviridae*. We find in total six candidate taxon levels two of which correspond to the species and genus ranks of the family. At these two levels, the six filovirus species and two genera officially recognized by ICTV, as well as a seventh tentative species for Lloviu virus and prototyping a third genus, are reproduced. DEmARC lends the highest possible support for these two as well as the four other levels, implying that the actual number of valid taxon levels remains uncertain and the choice of levels for filovirus species and genera is arbitrary. Based on our experience with other virus families, we conclude that the current sampling of filovirus genomic sequences needs to be considerably expanded in order to resolve these uncertainties in the framework of genetics-based classification.

## 1. Introduction

For a steadily growing number of viruses the genome sequence is the first and only information available. Because experimental characterization lags behind and is unlikely to be pursued for many viruses, comparative sequence analysis plays a central role in identifying commonalities and specifics between viruses. In this framework, researchers increasingly explore the usability of genetic sequences for virus classification. We have recently introduced a computational approach to hierarchically classify viruses of a family by relying solely on genetic data, coined DEmARC. Briefly, in DEmARC virus clusters are delimited by devising a threshold on the maximum intra-cluster (intra-taxon) divergence of viruses. This is done separately for each level of the hierarchical classification, all of which are selected using a cost function that measures the quality of virus clustering. The approach was extensively evaluated in a case study of picornaviruses [1]. Strikingly, the DEmARC-based picornavirus classification showed only few, but biologically notable, deviations from the ICTV taxonomy of the family *Picornaviridae* [2], the latter being developed by extensive efforts of expert picornavirologists who rely on various virus characteristics [3]. This analysis revealed important key parameters of DEmARC that distinguishes it from distance-based classification approaches and their applications of similar studies [4,5,6,7,8,9,10,11,12,13,14,15,16,17]. The DEmARC specifics include (i) the use of pairwise evolutionary distances (PEDs) instead of uncorrected p-distances, and (ii) a quantitative method to devise taxon levels and associated PED thresholds for virus clustering in a systematic and family-wide manner. We also reasoned that, in order to avoid a biased selection of genes/protein domains and to represent a virus genome as fully as possible, all family-wide conserved proteins must be used in a DEmARC-mediated analysis. As result, most incomplete genome sequences are excluded from the analysis. Since some of these differences are evolutionary-based, the DEmARC-based picornavirus classification enabled biological implications that are not (yet) available in taxonomy or through other approaches to virus classification. They include the prediction of known and currently unknown genetic diversity in the family and the proposed genetic separation of members of virus species [2]. So far, DEmARC was used to extensively revise the taxonomy of coronaviruses [18], and to propose the classification of two recently discovered insect nidoviruses [19,20] into the tentative new family “Mesoniviridae” [21]. In order to validate a general applicability of DEmARC in RNA virus taxonomy a systematic analysis of viruses from diverse families is most wanted.

In this study we sought to apply DEmARC to filoviruses, making it the first analysis of viruses with RNA genomes of negative polarity (ssRNA−). Filoviruses form the family *Filoviridae* of the order *Mononegavirales*, the latter combining all known ssRNA− viruses with non-segmented genomes [22]. Currently the filovirus genera *Marburgvirus* and *Ebolavirus*, which comprise one and five species, respectively, are recognized [23,24,25]. Additionally, a tentative genus “Cuevavirus” with a single species formed by Lloviu virus has been proposed [24]. Filovirus species are delimited using both phenotypic and genetic demarcation criteria including thresholds on percentage identity of full-length genomic sequences [24,25,26,27]. The filovirus genome of about 19 kb encodes seven structural proteins in separate open reading frames (ORFs). They include a nucleoprotein (NP), a spike glycoprotein (GP_1,2_), two matrix proteins (VP40 and VP24), a multi-domain protein (L) with RNA‑dependent RNA polymerase (RdRp) and methyltransferase function [28], an RdRp cofactor (VP35), and a transcriptional activator (VP30) [29]. Additionally, some filoviruses may encode lineage-specific proteins [30,31,32,33]. Marburg- and ebolaviruses are endemic to central Africa and the Philippines [26,27,34,35] while Lloviu virus was discovered in southern Europe [30]. Some filoviruses have been isolated from bats and domesticated pigs and can cause hemorrhagic fevers in primates with often fatal outcome. Since they comprise some of the most dangerous pathogens in the world, the taxonomy of filoviruses, especially at the species level, may have considerable practical implications.

## 2. Results and Discussion

**Figure 1:**
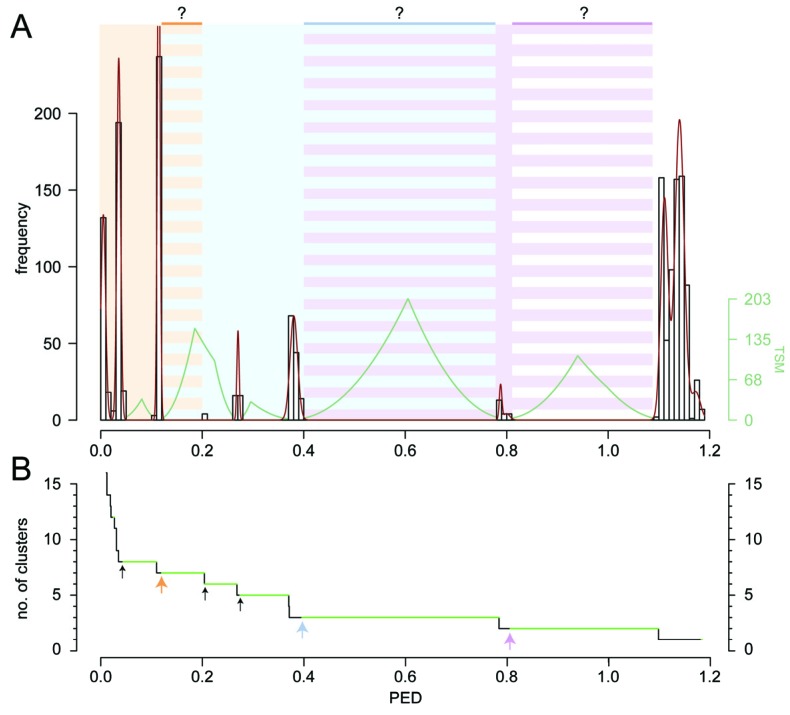
Filovirus-wide pairwise evolutionary distance (PED) distribution and thresholds for virus clustering. (**A**) Shown is a frequency distribution (white bins) of around 1,500 PED values, which estimate the average number of amino acid substitutions per site between each pair of viruses. A mixture model (red curve) was fitted to the PED distribution and used to calculate a threshold support measure (TSM, green). For details on threshold delineation see [1]. A threshold candidate (peak in the TSM measure) may be used to group the viruses into clusters in which virus pairs with a PED not exceeding the threshold join the same cluster. The three candidates with highest TSM scores were used to hierarchically group the 56 filoviruses at three levels comprising seven, three, and two clusters, respectively. Each of the thresholds is located within a continuous PED range for which no PED values are observed with the current filovirus sampling (striped background shading). PED values in these ranges (if sampled in the future) could be associated with either the classification level delimited by the respective threshold (intra-cluster distances) or with the next higher level (inter-cluster distances). The actual PED values of the thresholds are thus uncertain (bright-colored horizontal bars and question marks); for simplicity we selected the values (0.120, 0.396, and 0.806) that correspond to the smallest value within the respective PED range. (**B**) The change in the number of derived clusters with respect to the value of the PED threshold candidate is shown. For each threshold candidate a continuous PED range with optimal clustering cost of zero (no intra-cluster PED values exceed the threshold) is highlighted in green. The six threshold candidates considered in this study show the following PED ranges (from left to right): 0.044–0.109, 0.120–0.203, 0.204–0.267, 0.274–0.369, 0.396–0.783, and 0.806–1.097. The three threshold candidates used in (A) are indicated by colored arrows; three alternative PED thresholds for the species level are shown by black arrows. Note that another clustering (12 clusters) with zero cost was not considered in this study because of the marginal PED range and low TSM support of the associated threshold (PED of 0.025).

The dataset of this study was formed by 56 complete filovirus genome sequences that we downloaded into the Viralis platform [36] in February 2012. For these filoviruses, a concatenated multiple alignment of the seven proteins that are conserved family-wide was constructed and submitted to PED calculation. The resulting PED values are distributed non-uniformly along the range of 0 to 1.2 substitutions per amino acid position (Figure 1A). Using DEmARC we identified six distance threshold candidates, each associated with the optimal clustering cost of zero (Figure 1B) (see Experimental section). With this highest possible support all intra-cluster PED values would fall below the respective distance threshold, indicating that all clusters in a respective level may not be improved further (see [1] for technical details). This result suggests the use of all six thresholds for building a classification. However, the number of ranks below the family level is limited to three in virus taxonomy (subfamily, genus, and species). To satisfy this limitation, three thresholds must be selected by using additional criteria, e.g., biological properties. Within the DEmARC framework we used those thresholds that are associated with the highest threshold support measure (TSM) values. This use of TSM values is non-canonical: in DEmARC they are commonly used for the selection of PED ranges in which thresholds are further identified by local cost optimization if that is attainable (in situations where the optimal clustering cost of zero cannot be achieved; see [1] for technical details). In the current analysis of filoviruses, however, using each of the PED threshold candidates within the six PED ranges would result in a clustering cost of zero (Figure 1B). Thus, for each range we arbitrarily selected the smallest observed PED value within the range as the threshold value (colored arrows in Figure 1B). We note that the produced classification accommodates laboratory-introduced genetic variation in some sequences due to virus propagation in tissue culture before sequencing. The observed continuous ranges with zero PED frequency imply that the scale of this variation is small compared to the natural genetic variation even at the lowest level of the derived classification. 

**Figure 2:**
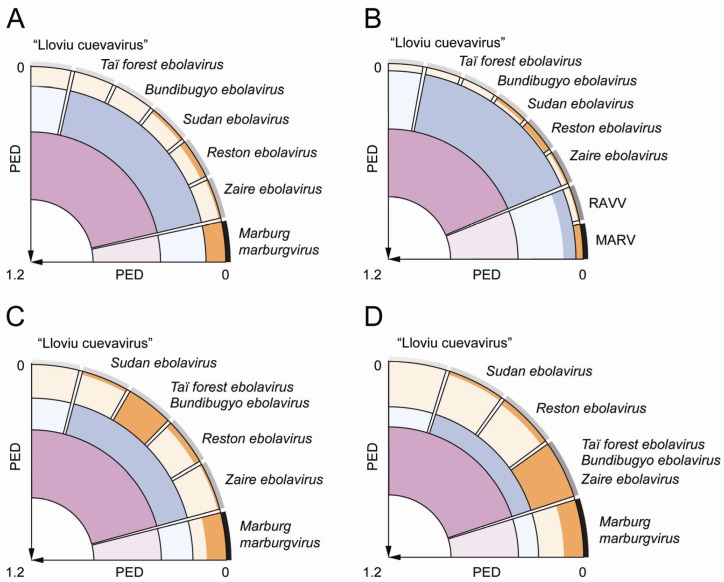
Genetics-based classification of filoviruses and alternative species groupings. A quadrant is used to visualize the classification of the 56 filoviruses with three hierarchical levels. The axes indicate intervirus genetic divergence (as PED) which increases linearly from the perimeter of the quadrant (zero PED) to its origin (maximum PED of 1.2). The three classification levels are highlighted using three basic colors (orange, blue, and purple). Each color exists in two shadings that highlight the limit on expected intragroup genetic divergence according to a distance threshold (soft shading) and the maximum observed intragroup genetic divergence (bright shading) of a cluster. (**A**) The genetics‑based filovirus classification using the three top-ranked PED thresholds. It comprises seven, three, and two clusters, respectively, at the three hierarchical levels. The seven orange clusters of the lowest level correspond to the official or tentative ICTV species of the family *Filoviridae* [25] and are indicated by names. The three blue clusters correspond to the official or tentative ICTV genera of the family *Filoviridae* (from left to right: “Cuevavirus”, *Ebolavirus*, and *Marburgvirus*); the two purple clusters correspond to supra‑generic taxa currently not recognized in the ICTV filovirus classification. Outside the quadrant, the relative density of virus sampling per ICTV species is shown as gray shadings from low (light) to high (dark) sampling, which is in the range of 1 to 34. (**B**) An alternative classification with eight instead of seven clusters at the lowest level. It was derived by using the alternative species threshold at PED = 0.044. (**C**) An alternative classification with six instead of seven clusters at the lowest level. It was derived by using the alternative species threshold at PED = 0.204. (**D**) An alternative classification with five instead of seven clusters at the lowest level. It was derived by using the alternative species threshold at PED = 0.274.

**Figure 3:**
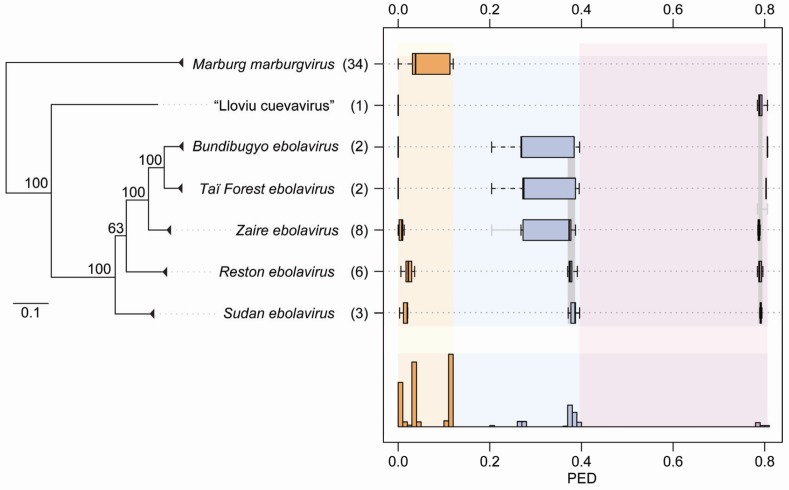
Intragroup genetic divergence of filoviruses. Box-and-whisker graphs are used to plot level-specific PED distributions for the seven clusters of the lowest classification level (bright-shaded orange, blue, and purple). The seven orange clusters correspond to the official or tentative ICTV species of the family *Filoviridae* [25] and are indicated by names; virus sampling per ICTV species is shown in brackets. The combined PED distributions of clusters of the second and third level are shown as gray box-and-whisker graphs. The expected range of level-specific PED values, bordered by two distance thresholds, is indicated by light-shaded background colors. The respective part of the PED distribution covering the full range of intragroup PED values of the three levels is show at the bottom. The ICTV species are grouped vertically according to a maximum likelihood phylogeny shown at the left. Internal nodes of the tree that correspond to ICTV species are collapsed (triangles); all of them have a bootstrap support value of 100.

The first selected threshold (PED of 0.120) results in seven clusters (Figure 1B) that match the official or tentative ICTV species of the family *Filoviridae*. These are species *Marburg marburgvirus* (comprising 34 virus sequences), species *Zaire ebolavirus* (8), species *Reston ebolavirus* (6), species *Sudan ebolavirus* (3), species *Taï Forest ebolavirus* (2), species *Bundibugyo ebolavirus* (2), and tentative species “Lloviu cuevavirus” (1). According to the second threshold (PED of 0.396), three clusters that match the official or tentative genera—*Marburgvirus*, *Ebolavirus*, and “Cuevavirus”—of the family are recognized (Figure 1B). The third threshold (PED of 0.806) joins viruses of the genus *Ebolavirus* and the tentative genus “Cuevavirus” into a single cluster while viruses of the genus *Marburgvirus* form the second cluster; these two clusters could be provisionally treated as tentative subfamilies. Hierarchical relationships of the 12 clusters according to the three applied distance thresholds are shown in Figure 2A. All delineated clusters form monophyletic lineages in the phylogeny of the 56 filoviruses (reciprocal monophyly) (Figure 3) when assuming the root to be closest to the branch leading to marburgviruses (which would correspond to midpoint rooting). The three threshold candidates not considered for classification (PED of 0.044, 0.204, and 0.274) would result in eight, six, and five clusters, respectively (Figure 1B). The clustering in eight clusters would split viruses of the species *Marburg marburgvirus* into two clusters formed by the RAVN and MARV lineage, respectively (Figure 2B). The clustering in six clusters would join viruses of the species *Taï Forest ebolavirus* and *Bundibugyo ebolavirus* into a single cluster (Figure 2C). The clustering in five clusters would join viruses of the species *Zaire ebolavirus*, *Taï Forest ebolavirus*, and *Bundibugyo ebolavirus* into a single cluster (Figure 2D). It would be reasonable to consider these eight-, six-, and five-cluster scenarios as alternative species groupings. On the other hand, it could indicate that the three taxonomic ranks below the family level may be insufficient to accurately classify the genetic diversity of filoviruses. In the DEmARC-mediated classification of coronaviruses we also observe numerous PED thresholds with zero clustering cost [37].

The PED threshold delineating the seven ICTV species is controlled by a single cluster, species *Marburg marburgvirus*, which shows by far the highest sampling (61% of all sequences) among all clusters of that level (Figure 2A and Figure 3). This cluster contributes the largest intracluster PED value (0.120) of that level compared to other clusters for which values of at most 0.036 (between viruses of the species *Reston ebolavirus*) are observed (Figure 3). These considerable differences in the divergence of viruses from different filovirus species can be rationalized through two contrasting explanations that both exploit possible effects of biased virus sampling on the classification. First, the observed differences could be a result of the relatively poor sampling of virus sequences from the five ebolavirus and the “cuevavirus” species. Once sampling is improved, viruses from these species may show a genetic divergence comparable to marburgviruses. Alternatively, future improved virus sampling might show that the five ebolavirus species recognized by ICTV actually form a single species. The full PED range from 0 to around 0.4 (Figure 1A) might then be populated which would merge viruses of the five ebolavirus species into a single cluster. This would result in three species clusters in total corresponding to the three ICTV genera of the family *Filoviridae*. Consequently, ebolaviruses and “cuevaviruses” would form a single genus (in addition to the genus *Marburgvirus*) as suggested by the PED threshold of 0.806 (Figure 1 and Figure 3). The above scenarios are just few from many possible (e.g., see the alternative species groupings in Figure 2B–D) and illustrate the current uncertainty about filovirus classification. The three-species scenario seems to be conceivable when comparing the filovirus PED distribution with that of the well-sampled family *Picornaviridae* with up to 260 available sequences per species (more than 1,200 sequences in total distributed among 38 species clusters) [1]. In the high-sampling case of picornaviruses, no PED values with zero frequency are observed which suggests that the current sampling of filovirus genome sequences may strongly underestimate the natural genetic diversity in the family. Expanded virus sampling might also lead to a better differentiation of filovirus proteins by evolutionary criteria. Currently, all seven proteins are conserved family-wide among known filoviruses, which may change for this family in the future when most diverged viruses are separated by (much) larger genetic distances, as we already observe for picornaviruses and many other families. This development would also affect the choice of proteins by DEmARC and, consequently, the resulting classification. Furthermore, the PED threshold values are likely to change in the future, even if the underlying virus clusters will be stable, given the large PED ranges they currently represent (Figure 1B). Thus, a definite decision about number and virus composition of filovirus species and genera as well as stable demarcation thresholds will only be possible if the sampling of filovirus genome sequences is expanded in the future.

We note that the use of pairwise sequence similarities is becoming increasingly popular for assisting decision-making in virus taxonomy (see the Introduction for literature). On the other hand, there is the current paradigm that the use of a single (e.g., genetic) criterion is insufficient for the demarcation of viral taxa [38]. In our opinion, a fully genetics-based classification provides a promising and meaningful foundation for virus taxonomy. Indeed, the genome is the principal carrier of genetic information and heredity; and it was already acknowledged that virus taxonomy should reflect the evolutionary history of viruses [39] which can be reconstructed from genetic data. However, analyzing the evolutionary record in genomes remains a challenging task with many parameters to define and is dependent on the amount of available genetic information (see above). Consequently, the technical implementation and associated choices made during an analysis (e.g., using a relatively simplistic measure of pairwise distances) may affect the quality of genetics-based classification. Different directions of future research efforts are conceivable, ranging from the development of improved evolutionary models for the calculation of genetic distances [40] to entirely different techniques of utilizing the genetic information for virus classification [41,42]. As the development and application of DEmARC illustrates, this line of research offers a possibility of tackling the virus classification problem in a systematic manner.

In the DEmARC framework, an upper limit on intra-species genetic divergence is imposed on loci encoding the family-wide conserved proteins. Consequently, when there is a strong support for species, viruses of the same species are genetically separated from viruses outside the species in these loci. For picornaviruses with their RNA genomes of positive polarity (ssRNA+), this separation may be promoted by mutation and limited through homologous recombination [2,43,44], as we argued [2]. Recombination among ssRNA− viruses was estimated to be generally rare [45] and this was explained by major differences in the replication cycle (due to the negative polarity of genomes) compared to ssRNA+ viruses, which limits the template-switching ability of the RdRp through rapid packaging of the genomic RNA with ribonucleoproteins [46]. However, homologous (intrasegmental) recombination can occur as was found for different ssRNA− viruses [47,48,49] including the prototype ebolavirus, Ebola virus [50]. The frequency with which both ssRNA+ and ssRNA− viruses recombine in nature remains to be shown but it may not be uncommon [51], and currently available recombination detection tools were shown to underestimate this frequency in certain situations [49]. Ultimately, both the rate of homologous recombination among RNA viruses in nature and the model of genetic separation of virus speciation should be probed experimentally. 

## 3. Experimental Section

The seven filovirus proteins that are conserved in all known members of the family (NP, VP35, VP40, GP_1,2_, VP30, VP24, and L) were aligned separately at the amino acid level using the program Muscle version 3.52 [52] followed by manual correction. The seven protein alignments were concatenated to form a single alignment of 5,143 positions with a gap content of 5.5%. To estimate the genetic similarity between virus pairs, PED values were calculated on this concatenated alignment using the program Tree-Puzzle version 5.2 [53]; the WAG amino acid substitution matrix was applied [54]. Proteins that are not conserved family-wide, like sGP and ssGP of ebolaviruses and “cuevaviruses” or certain hypothetical proteins encoded by the anti-sense genomic RNA, were not included in the calculation of PED values. For these proteins, an accurate estimation of genetic divergence may be approached only for the selected filoviruses that encode these proteins. The distribution of all PED values was partitioned into intra-rank and inter-rank ranges using a systematic approach implemented in DEmARC [1]. This partitioning is achieved through the inference of PED thresholds below which two viruses are grouped together. We refer to the resulting virus groups as “clusters” in the context of genetic classification by DEmARC. A cluster might correspond to a viral taxon officially recognized by ICTV.

A derivative of the multiple alignment used for PED calculation, from which strongly conserved blocks [55] (in total 3,522 alignment positions, 68.5%) have been extracted by BAGG [56], formed the dataset for unrooted tree reconstruction by PhyML version 3.0 [57]; the WAG amino acid substitution matrix was applied [54]; support for internal nodes was obtained through a non-parametric bootstrap analysis with 100 replicates.

## 4. Conclusions

DEmARC offers a systematic and quantitative framework for virus classification that utilizes genome sequences, the only information available for a growing number of viruses. The striking agreement on species and genus taxa between the DEmARC-mediated filovirus classification of this study and the taxonomy of the family *Filoviridae* could be considered a cross-validation for both. However, we note that this classification is one of many equally strongly supported classifications, as DEmARC identified in total six potential taxon levels. Each of these taxon levels is associated with a large continuous range of PED values that are not sampled (yet). Consequently, we conclude that the current coverage of the natural genetic diversity of filoviruses is limited and needs to be considerably expanded, also concerning hosts not sampled so far, in order to gain certainty about both filovirus taxa and levels.
